# A Simplified Method for Patterning Graphene on Dielectric Layers

**DOI:** 10.1021/acsami.1c09987

**Published:** 2021-07-30

**Authors:** Håkon I. Røst, Benjamen P. Reed, Frode S. Strand, Joseph A. Durk, D. Andrew Evans, Antonija Grubišić-Čabo, Gary Wan, Mattia Cattelan, Mauricio J. Prieto, Daniel M. Gottlob, Liviu C. Tănase, Lucas de Souza Caldas, Thomas Schmidt, Anton Tadich, Bruce C. C. Cowie, Rajesh Kumar Chellappan, Justin W. Wells, Simon P. Cooil

**Affiliations:** † Center for Quantum Spintronics, Department of Physics, Norwegian University of Science and Technology (NTNU), NO-7491 Trondheim, Norway; ‡ Department of Physics, Aberystwyth University, Aberystwyth SY23 3BZ, United Kingdom; § School of Physics & Astronomy, Monash University, 1 Wellington Rd., Clayton, Victoria 3800, Australia; ∥ School of Physics, HH Wills Physics Laboratory, University of Bristol, Tyndall Avenue, Bristol BS8 1TL, United Kingdom; ⊥ School of Chemistry, University of Bristol, Cantocks Close, Bristol BS8 1TS, United Kingdom; # Department of Interface Science, Fritz-Haber-Institute of the Max-Planck Society, Faradayweg 4-6, 14195 Berlin, Germany; ∇ Australian Synchrotron, 800 Blackburn Rd., Clayton, Victoria 3168, Australia; ○ Semiconductor Physics, Department of Physics, University of Oslo (UiO), NO-0371 Oslo, Norway

**Keywords:** graphene, patterned growth, electrical decoupling, photoelectron spectroscopy, PEEM, LEEM, NEXAFS

## Abstract

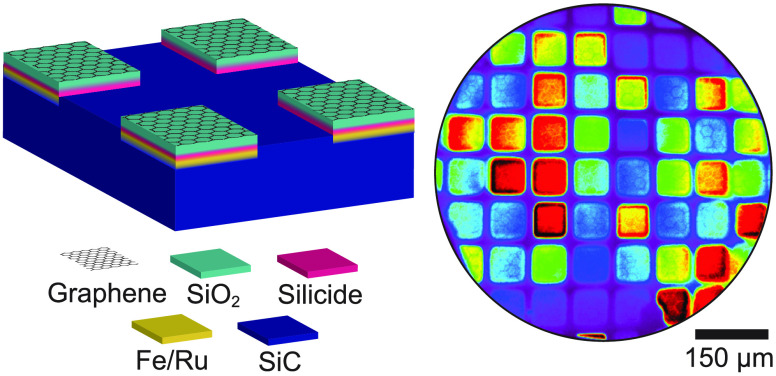

The large-scale formation of patterned, quasi-freestanding graphene structures supported on a dielectric has so far been limited by the need to transfer the graphene onto a suitable substrate and contamination from the associated processing steps. We report μm scale, few-layer graphene structures formed at moderate temperatures (600–700 °C) and supported directly on an interfacial dielectric formed by oxidizing Si layers at the graphene/substrate interface. We show that the thickness of this underlying dielectric support can be tailored further by an additional Si intercalation of the graphene prior to oxidation. This produces quasi-freestanding, patterned graphene on dielectric SiO_2_ with a tunable thickness on demand, thus facilitating a new pathway to integrated graphene microelectronics.

## 1 Introduction

The extraordinary properties and success of graphene in prototype electronic platforms have led to numerous synthesis routes and processing methods for patterning the material. While isolation of graphite layers by mechanical exfoliation led the field in characterizing the material properties,^1,2^ using exfoliation for device fabrication suffers from a low yield and a lack of scalability.^3^ Scalable methods that do not rely on exfoliation are therefore attractive.

Chemical vapor deposition (CVD) on metal^4,5^ and semiconductor^6,7^ substrates or high-temperature annealing of SiC^8−10^ has gained the most traction for producing high-quality graphene films. A large number of routes for further processing are now available, such as photolithography,^11,12^ electron beam lithography,^13,14^ scanning probe lithography/etching,^15,16^ and direct laser lithography.^17−19^ However, these methods each have their own disadvantages, e.g., growth on a metal substrate requires the graphene to be transferred and patterning induces defects/contaminants that reduce device efficacy.^20,21^

A promising transfer-free method utilizes a transition-metal catalyst (Cu or Ni) deposited directly onto an oxide layer on a Si wafer.^22,23^ Graphene is then formed by annealing in the presence of carbon, and the metal film is subsequently removed chemically, leaving the graphene in direct contact with the dielectric material. Although this method shows great potential, metal contamination remains a major issue.^24^ An alternative approach for removing metal from the graphene has been demonstrated,^25^ as has a method for adding a dielectric layer under the graphene after growth.^26^ Both modify the graphene–substrate interaction, which is known to impact the electronic properties of graphene.^27−30^ Although there is no single method that suits all device applications, there is a desire for transfer-free methods that produce graphene free of contaminants and with control of the substrate interaction, directly on dielectric surfaces. In this work, we demonstrate transfer-free, patterned graphene structures on SiC, with optional decoupling by forming SiO_2_ at the graphene–semiconductor interface.

## 2 Methods

In our previous work, few-layer graphene on transition-metal silicide was prepared according to the metal-mediated approach as described elsewhere.^31,32^ Thin films of Fe or Ru (1–2 nm) on 6H-SiC(0001) were thermally activated by annealing to temperatures of 600–700 °C for a short duration. This triggered transition-metal silicide formation at the interface and the associated liberation of carbon reconstructing into graphitic (sp^2^) layers on the surface. Following the growth, all samples were subsequently annealed to higher temperatures (*T* > 700 °C) to diffuse the metal into the bulk of the underlying SiC substrate.^25,32^ This left the graphene layers supported directly on a thin film of Si.

To demonstrate the possibility of patterning graphene we followed the above approach, but now with predefined metallic regions (prior to graphene growth) in regular arrays of squares or circles by means of a simple shadow mask. The metals Fe and Ru were deposited under ultrahigh vacuum (UHV) onto chemically and thermally cleaned 6H-SiC substrates through solid masks made of a Mo foil with openings of 50 and 500 μm that were placed in proximity (<0.3 mm) to the sample surface.

## 3 Results and Discussion

**Figure 1:**
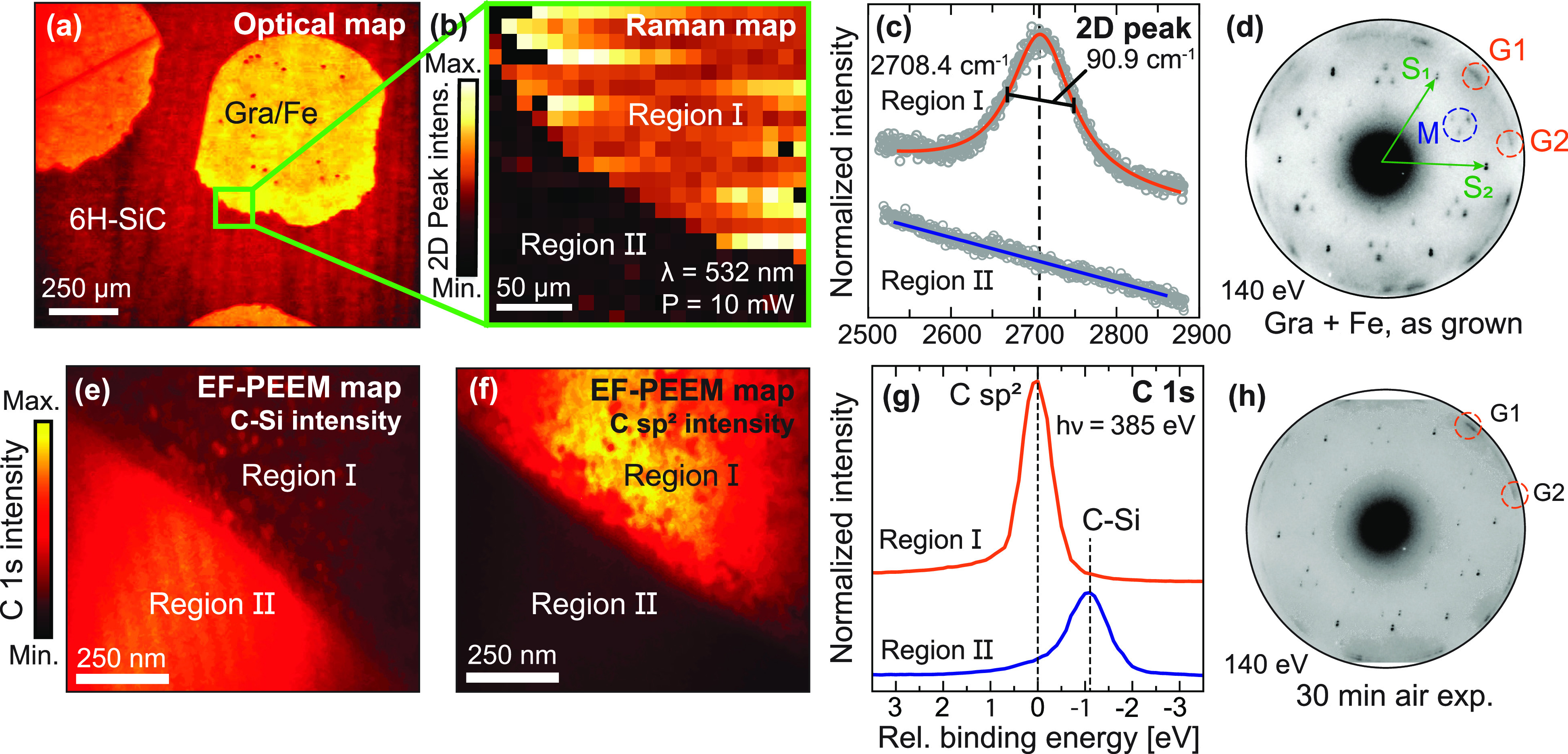
Localized graphene growth on top of patterned Fe. (a) Optical micrograph (artificially colored) showing circular regions of graphene on Fe patterned on 6H-SiC(0001). (b) Spatially resolved Raman showing the intensity of the 2D graphene peak near the edge of one of the patterned regions. (c) Intensity and full width at half-maximum (FWHM) of the 2D Raman peak recorded from the two different spatial Regions I and II in panel 1b. (d) Small-area low-energy electron diffraction (μ-LEED) pattern of Fe-mediated, patterned graphene on 6H-SiC(0001) grown at 600 °C. The threefold symmetric diffraction pattern of the substrate is described by vectors **S**_1_ and **S**_2_. Two distinct rotational domains of graphene (G1, G2) appear at higher |**k**|, with ≈15 and 45° relative to the SiC. Additional spots (M) can be assigned to remnants of Fe beneath the graphene. (e, f) EF-PEEM measurements of a patterned region recorded at two different binding energies corresponding to C–Si and C sp^2^ bonding, respectively. (g) C 1s core level extracted from Regions I and II in the EF-PEEM. The topmost trace (Region I) clearly demonstrates the confinement of graphene within the pattern, while the bottom trace (Region II) shows C–Si bonding characteristics for the SiC substrate. (h) μ-LEED pattern of the Fe-mediated graphene from panel 1d after 30 min. air exposure: G1 and G2 are still visible and thus indicate that the graphene is stable when exposed to air.

Selectively grown graphene exclusively on top of Fe islands is demonstrated in Figure 1, using Raman spectroscopy, energy-filtered photoemission electron microscopy (EF-PEEM), and low-energy electron diffraction (LEED). In the Raman spectrum, a distinctive “2D peak” from sp^2^ hybridized carbon is observed at 2708.4 cm^–1^ (Figure 1c). The full width at half-maximum (FWHM) of the peak is 90.9 cm^–1^, suggesting that either mono- or bilayer graphene has been created.^33^ In Figure 1b, the spatial distribution and intensity of this peak are plotted, showing two distinctly different areas that indicate patterned graphene formation. Intensity from the graphene is predominantly found inside the Fe pattern (Region I), while negligible amounts can be seen from the nonmetalized SiC substrate (Region II).

The spatially resolved C 1s core level intensity from a similarly prepared sample is shown in Figure 1e, 1f. Distinct chemical components from two separate regions can be distinguished (Figure 1g). Within the Fe pattern (Region I) an asymmetric peak shape appears at a binding energy of 284.5 eV, characteristic for sp^2^ bonded carbon.^34,35^ Outside the pattern (Region II) a symmetric peak from the C–Si bonds in SiC appears at 1.1 eV lower binding energy.^8^ The spatially resolved Raman and EF-PEEM thus demonstrate that graphene forms only within the metalized regions.

The crystalline quality of the graphene formed is demonstrated from the small spot (1.5 μm) low-energy electron diffraction (μ-LEED) pattern in Figure 1d. Twelve spots appear at ≈15 and 45° rotation relative to the unreconstructed (1 × 1) SiC phase described by vectors **S**_1_ and **S**_2_. These twelve spots have a |**k**| = 2.50 Å^–1^, i.e., within ±2% of what is expected for pristine graphene layers^36^ and are thus interpreted as two predominant graphene rotational domains, G1 and G2. Additional spots (M) are also observed that likely originate from an underlying bcc(110) lattice constrained by the hexagonal 6H-SiC(0001) surface. Similar features have been reported for Fe thin films on hexagonal surfaces^37,38^ and are removed at a later stage.^32^

**Figure 2:**
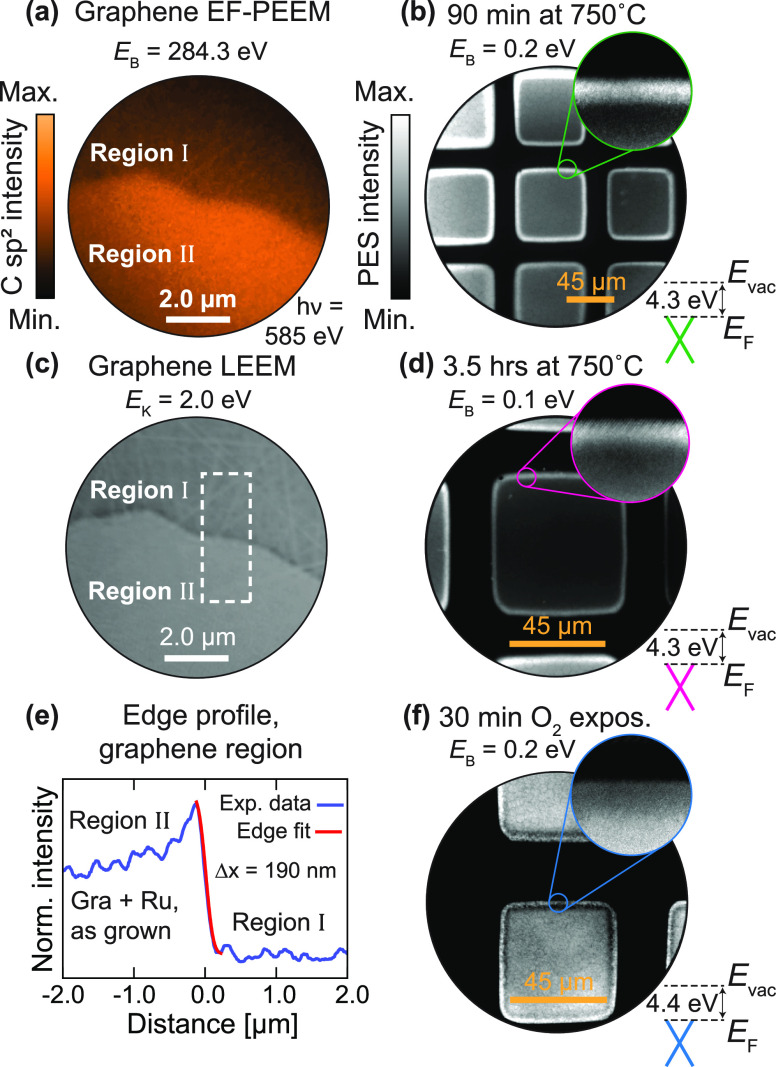
EF-PEEM and LEEM of patterned graphene structures. (a) EF-PEEM micrograph from a sample of 6H-SiC patterned with Ru (Region II) and annealed to 800 °C. The sample is probed at the graphene binding energy, confirming that the graphene formed is confined within the metalized region. (c) LEEM micrograph of the same region probed for electron kinetic energy *E*_k_ = 2.0 eV. (e) Fitted intensity profile of the patterned graphene edge taken from within the dashed white area in panel 2b. The error function fit (in red) reveals a patterning resolution of Δ*x* = 190 nm. (b,d,f) EF-PEEM micrographs showing patterned 2 nm Ru on SiC annealed to 750 °C for increasing the time duration to form graphene at the surface (b, d), and finally after 30 min of exposure to air (f). Each micrograph has been extracted for binding energies *E*_B_ close to the Fermi level *E*_F_. The average work function (WF) within the squares is indicated in the lower right corner of each subpanel.

In our earlier work, thermalized thin films of Ru on 6H-SiC have been shown to produce graphene of a similar quality to those mediated by thin films of Fe.^32^ The spatial distribution of C 1s core level signal from this system is resolved in Figure 2a. Again a distinct, asymmetric line shape appears only within the growth region at 284.3 eV binding energy, verifying that graphene forms exclusively within the patterned Ru.

The lateral resolution of the patterning can be ascertained from the sharpness of the boundary between the graphene region and the SiC substrate, as shown in the low-energy electron microscopy (LEEM) image in Figure 2c. A fitted intensity profile along the edge gives a patterning resolution of 190 nm (Figure 2e). We suspect that this estimate is limited by the bright and dark fringes in the image, resulting from a sudden change in electrostatic potential and work function at the boundary,^39−41^ rather than by the abruptness of the patterning.

When annealed for a longer duration, the patterned geometry and the photoemission signal from the graphene in each growth region are well preserved, as shown in Figure 2b, 2d. However, a higher photoemission intensity is seen around the edges of each structure. This may suggest locally enhanced nucleation and associated growth rate, as reported for epitaxial graphene on SiC^42−44^ and other 2D materials.^45^

The chemical stability of the patterned graphene was tested by exposing both systems to air for 30 min. In Figure 1h, a LEED pattern from the same Fe-mediated graphene as in Figure 1d is shown after 30 min of exposure to air and a reintroduction to UHV. The two patterns are comparable, indicating that the surface graphene is stable to the exposure. However, the background intensity of the pattern is seen to increase.

Chemical stability is also demonstrated by the small variation in work function between the surfaces in Figure 2b, 2f, and 2. The graphene once formed (Figure 2b, 2d) has a work function of 4.30 eV, and after air exposure (Figure 2f), the work function increases slightly by 100 meV. Given the chemical inertness of graphene already established from our LEED measurements and in the literature,^46−48^ this small energy increase hints at a change in the potential at the graphene interface. Furthermore, the intensity of secondary electrons (SEs) near the low-energy cutoff increases by roughly 1 order of magnitude. This can be explained by the longer inelastic mean-free path of photoelectrons associated with the wider band gap of SiO_2_.^49^ Together with the higher background intensity seen from the LEED (Figure 1h), these changes indicate silicon oxide formation at the graphene–substrate interface.

To further explore the possibility of electrically decoupling the graphene, insulating SiO_2_ was grown directly at the semiconductor–graphene interface. To determine how the oxide growth would affect the patterned graphene, spatially resolved, high-resolution X-ray photoemission spectroscopy (XPS) and near-edge X-ray absorption fine structure (NEXAFS) measurements were performed both inside and outside of the graphene growth regions. Note that to perform spatially resolved, high-resolution spectroscopy like this, particularly the NEXAFS, samples of metal-mediated graphene were produced with a patterning scale of 5 mm to compensate for the larger spot size of the photoexcitation.

**Figure 3:**
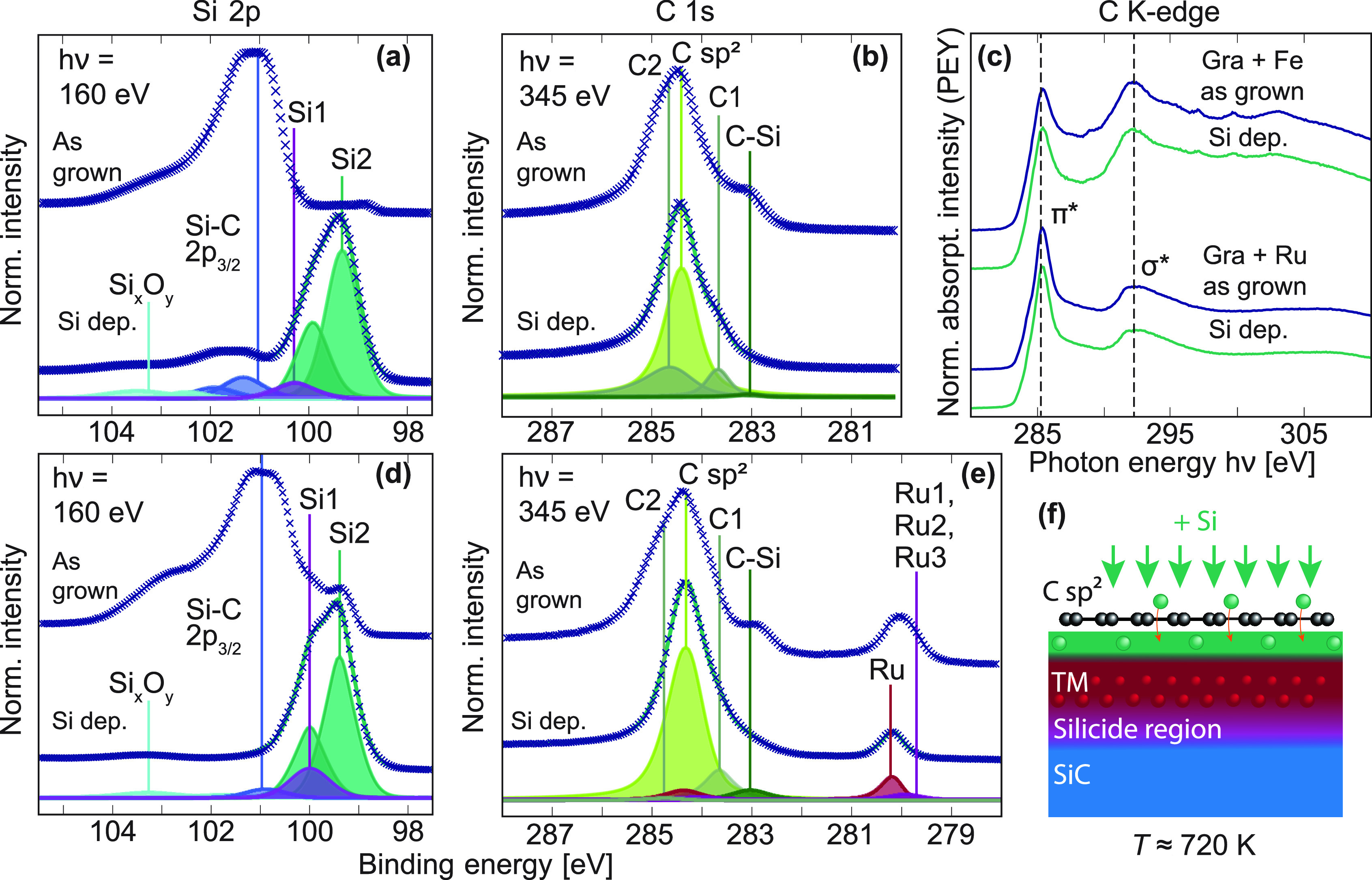
Spatially resolved XPS and NEXAFS demonstrating Si intercalation of metal-mediated graphene. (a, b) Surface-sensitive core levels Si 2p and C 1s from graphene growth using Fe, obtained before and after intercalation of 0.6 nm Si. The C 1s, consisting of graphene (C sp^2^), substrate (C–Si), and two surface-related signals (C1, C2), is stable during the Si treatment. Newfound Si from the intercalation (Si2) can be distinguished from the silicide (Si1), substrate (Si–C), and oxide (Si*_x_*O*_y_*) components. (d, e) Surface-sensitive core levels C 1s and Si 2p from graphene growth, now using Ru, before and after intercalation of 0.6 nm Si. The deconvolved core levels from the treated surface reveal similar features as for the Fe-mediated system. (c) Near-edge X-ray absorption fine structure (NEXAFS) measurements of the carbon *K*-edge of graphene grown using either Fe or Ru, before and after Si intercalation. Both systems were excited with linearly polarized light at grazing (θ ≈ 20°) incidence relative to the sample plane. (f) Schematic demonstrating the intercalation of Si adsorbates between graphene and its underlying growth substrate.

An oxide layer was grown by stepwise intercalation of Si and O_2_ as previously demonstrated for CVD graphene grown on transition-metal substrates.^26^ In the first step, patterned graphene was subjected to Si atoms at a flux of 0.15 Å/min for 40 min while being heated to 450 °C. Figure 3 shows the change induced to the carbon *K*-edge and the Si 2p and C 1s core levels for Fe- and Ru-mediated graphene samples. During deposition, the intensities and shapes of the 1s → π* excitations (Figure 3c) and the graphene core levels at 284.4 eV (Figure 3b, 3e) are well preserved. In contrast, signals from the substrate (C-Si) and Ru 3d_5/2_ (Ru, Ru1-Ru3) are attenuated strongly. New and prominent peaks (Si2) appear at lower binding energies relative to the surface Si–C and silicide (Si1) in the Si 2p region (Figure 3a, 3d). The preservation of the 1s → π* resonance, the stable graphene core level signal, and the mentioned attenuation of the Ru and SiC intensities, therefore, suggest that the added Si has intercalated the graphene layers without forming clusters on the surface.

**Figure 4:**
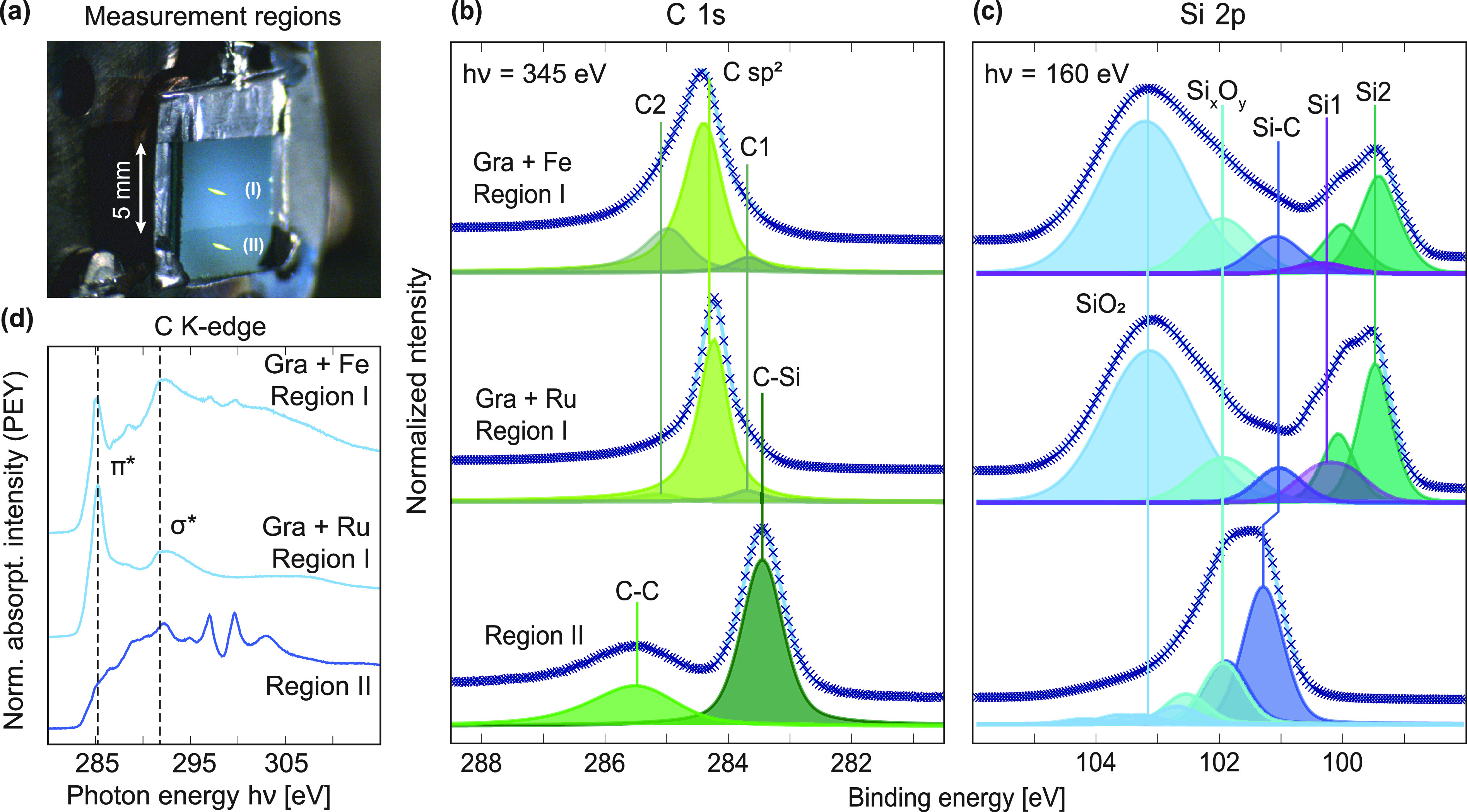
Spatially resolved NEXAFS and XPS of graphene after the growth of underlying SiO_2_ layers. (a) Photograph showing two distinct regions, one exposed (I) and the other shadowed (II) during metallization, graphene growth, and Si intercalation. The two yellow areas indicate the positions and size of the photoexcitation light spot used during XPS and NEXAFS data acquisition. (b, c) Surface-sensitive C 1s and Si 2p core levels after exposure to 200 mbar oxygen while being heated to 300 °C. The same core levels but from the bare SiC substrate are shown for comparison. (d) Grazing incidence (θ ≈ 20°) NEXAFS of Si-intercalated epitaxial graphene grown using Fe or Ru after subsequent oxidation of the underlying Si. NEXAFS from the bare SiC substrate (Region II) is also shown.

Next, the samples were heated to 300 °C while being exposed to O_2_ at 200 mbar to trigger the oxidation of the freshly intercalated Si. The shadow masks were then removed and the samples reintroduced to UHV. Spectroscopy measurements were repeated within the patterned graphene growth regions and on the bare and adjacent substrate for comparison (Figure 4). The photograph in Figure 4a shows the two regions under investigation, with a distinct boundary between them indicating the previous position of the now-removed shadow mask (Region II). The yellow spots indicate both the size and positions of the beam during photoexcitation(s).

With O_2_ exposure, a broad, new feature appears in the Si 2p signal (Figure 4c) of Region I at around 103 eV. A similar peak at 103.6 eV has previously been assigned to the Si^4+^ of SiO_2_.^50^ The formation of SiO_2_ in our data is furthermore supported by the loss of signal from the previously added Si, as seen by the strong attenuation of the Si2 signal. Comparing the Si 2p core levels from inside and outside the growth regions, the new SiO_2_ feature does not appear in Region II but rather a small tail of intermediate oxide states (Si*_x_*O*_y_*).^51,52^

As expected, no signatures of graphene can be observed in the region previously shadowed by the mask (Region II). The NEXAFS shows no π* resonance, and the only C 1s signals present are those corresponding to the C–C and C–Si bonds of the SiC surface.^53−56^ Inside the pattern (Region I), the graphene does not show any signs of oxidation: the π* resonances of the NEXAFS appear to be unchanged, and the energies, shapes, and intensities of the graphene core levels are well preserved. In Figure 4b, only a small redistribution of intensity can be seen between the surface C1 and C2 peaks. The characteristic Ru 3d_5/2_ signal from the Ru-mediated graphene system can no longer be distinguished at this stage. With the presence of SiO_2_ verified from the Si 2p signal, the robustness of the graphene peaks suggests that the graphene is now supported directly on top of the SiO_2_.

## 4 Conclusions

The demonstrated concepts of transfer-free and patterned graphene formation directly on dielectric thin films is an exciting development that allows structured graphene to be defined in a simple and straightforward manner with minimal extra processing required. Transition metals Fe and Ru can be used interchangeably to predefine the growth regions, and at moderate temperatures (600–700 °C), both will yield high-quality graphene that is robust against subsequent exposure to air. An underlying SiO_2_ layer can easily be formed through stepwise intercalation of Si and O_2_, in principle yielding oxides with precise thicknesses by controlling the dosage of each of the two constituents. Hence graphene–dielectric–semiconductor heterostructures with tailored and tunable oxide thicknesses can be produced. This simplistic approach is suitable for producing some of the building blocks for graphene-based device applications that rely on a semiconducting substrate/body, such as graphene-based field-effect transistors (GFETs)^57,58^ and radiation sensors.^59^ Our results thus demonstrate the principle and feasibility of transfer-free growth of graphene on dielectric, which may open up avenues for integrating the techniques presented into the established framework of semiconductor device processing.

## Supporting Information Available

Description of the TOC figure, a schematic outlining the growth procedure, and relevant experimental parameters for the results presented; and additional data outlining the Raman analysis, the metal diffusion into SiC, and comparative NEXAFS and XPS core levels from selected stages of the experiment (PDF)
