# Supplementary material for: A Simplified Method for Patterning Graphene on Dielectric Layers
Source: ACS Appl Mater Interfaces. 2021 Jul 30;13(31):37510–6. doi: 10.1021/acsami.1c09987 (PMC8365599; doi:10.1021/acsami.1c09987)
Supplement: Supplementary file 1 — am1c09987_si_001.pdf [file am1c09987_si_001.pdf]

Supporting Information: A Simplified Method for Patterning Graphene on Dielectric Layers

Håkon I. Røst,[*,†] Benjamen P. Reed,[‡,△] Frode S. Strand,[†] Joseph A. Durk,[‡] D. Andrew Evans,[‡] Antonija Grubišić-Čabo,[¶,∇] Gary Wan,[§] Mattia Cattelan,[‖,††] Mauricio J. Prieto,[⊥] Daniel M. Gottlob,[⊥] Liviu C. Tănase,[⊥] Lucas de Souza Caldas,[⊥] Thomas Schmidt,[⊥] Anton Tadich,[#] Bruce C.C. Cowie,[#] Rajesh Kumar Chellappan,[†] Justin W. Wells,[†,@] and Simon P. Cooil[*,‡,@]

†*Center for Quantum Spintronics, Department of Physics, Norwegian University of Science and Technology (NTNU), NO-7491 Trondheim, Norway.*

‡*Department of Physics, Aberystwyth University, Aberystwyth SY23 3BZ, United Kingdom*

¶*School of Physics & Astronomy, Monash University, 1 Wellington Rd., Clayton, Victoria 3800, Australia.*

§*School of Physics, HH Wills Physics Laboratory, University of Bristol, Tyndall Avenue, Bristol, BS8 1TL, United Kingdom*

‖*School of Chemistry, University of Bristol, Cantocks Close, Bristol, BS8 1TS, United Kingdom*

⊥*Department of Interface Science, Fritz-Haber-Institute of the Max-Planck Society, Faradayweg 4-6, 14195, Berlin, Germany*

#*Australian Synchrotron, 800 Blackburn Rd., Clayton, Victoria 3168, Australia.*

@*Semiconductor Physics, Department of Physics, University of Oslo (UiO), NO-0371 Oslo, Norway*

△*Present address: National Physical Laboratory (NPL), Hampton Road, Teddington, TW11 0LW, UK.*

∇*Present address: KTH Royal Institute of Technology, Applied Physics, Hannes Alfvéns väg 12, SE-114 19 Stockholm, Sweden.*

††*Present address: Elettra Sincrotrone Trieste, s.s. 14 - km.163,5 in Area Science Park, Basovizza, Trieste, 34149, Italy*

E-mail: hakon.i.rost@ntnu.no; scooil@icloud.com

Table of Contents (TOC) Graphic

The TOC graphic contains: (left) a schematic showing the final stage of the growth as described in more detailed in the following section, and (right) an artificially colored PEEM micrograph of patterned graphene on Ru, based on the photoemission yield near the low-energy secondary electron cutoff (SEC). The color scale of the micrograph as been chosen to amplify small variations in photoemission intensity between the structures.

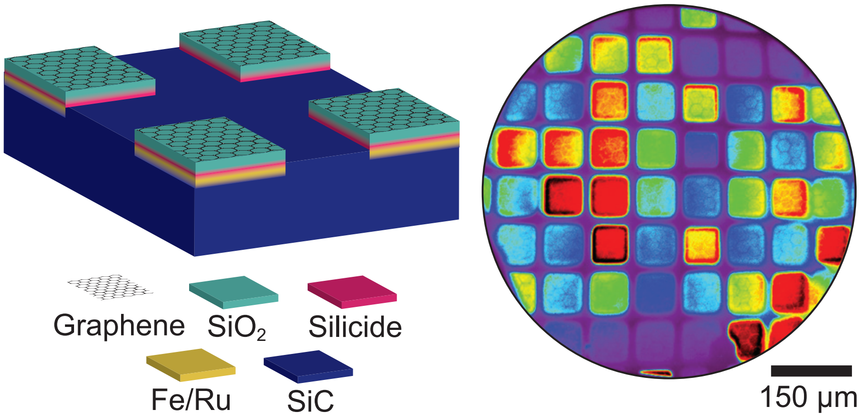

Figure S1: TOC graphic

Sample Preparation

The preparation of patterned graphene structures on SiO_2 is outlined in Figure S2. Graphene on SiC was grown according to the metal-mediated approach, utilizing thermally activated thin films of transition metals (TMs) Fe or Ru on top of HF etched and thermally cleaned 6H-SiC(0001) surfaces (see Refs. 1–3). The metal thin films were deposited through finely meshed, (50 and 500 µm) shadow masks, forming patterned metal islands with thicknesses 1-2 nm on the clean SiC surfaces (step I). These metal treated regions served as growth sites for graphene upon thermal activation to temperatures 600-700 °C, confining the lateral spread of graphene to within the boundary of each island (step II). X-ray photoelectron spectroscopy (XPS) measurements of graphene formation on Ru/SiC are shown in FIGs. S3e and S3f.

Upon formation the samples were heated to higher temperatures ($T > 700\,^{\circ}$C) for longer durations to diffuse the transition metal into the SiC substrate (step III). Finally, the samples were either i) exposed to atomic Si for 40 minutes at a rate of 0.15 Å/ min while being heated to 450 °C and then exposed to air, or ii) exposed to air directly (step IV). All samples were finally reintroduced to vacuum and gently annealed to 300 °C to desorb any contaminants from the air exposure.

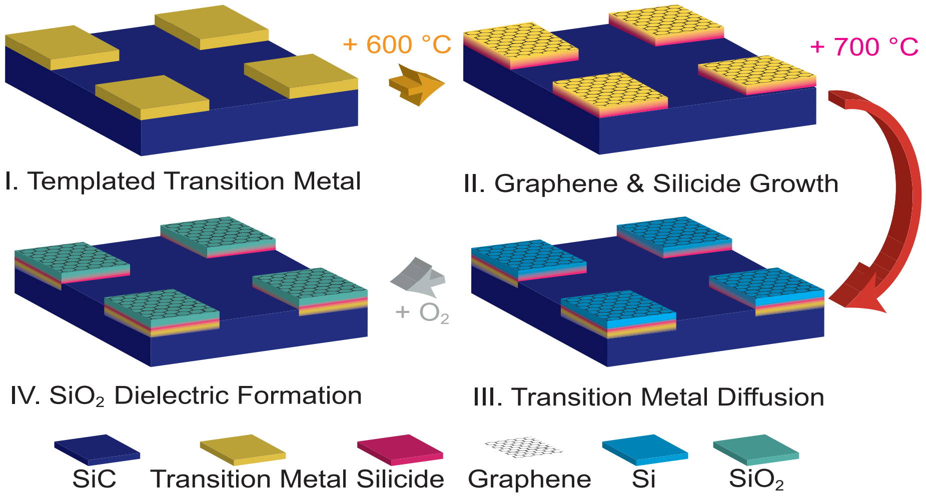

Figure S2: A Schematic overview of the four growth steps used to form patterned graphene structures on SiO_2.

Small-Spot EF-PEEM, LEEM and LEED Measurements

In order to assess the selectivity of graphene growth on top of the transition metal, samples patterned with Fe or Ru were studied before and after the thermal treatments using spatially resolved electron microscopy, electron diffraction and energy-resolved photoemission microscopy. At the UE49-PGM-SMART endstation of BESSY II, Helmholtz-Zentrum Berlin, Germany, graphene was prepared on SiC covered with 500 µm diameter islands of Fe or Ru thin films as described. Inside the graphene growth regions, energy-filtered PEEM (EF-PEEM) measurements of the C 1s, Fe 2p and Ru 3d core levels were performed at 300 K.

For the double aberration-corrected measurements of the C 1s shown in the main text, the overall energy resolution was 540-620 meV.

The lateral resolution of the patterned graphene was determined from the edge profile between the growth region and the SiC substrate. The region close to the edge was measured using low-energy electron microscopy (LEEM) with an electron kinetic energy $E_K = 2.0$ eV and fitted using an error function. From the optimized fit the standard deviation of the corresponding Gaussian was extracted and used to determine the full width at half maximum (FWHM) of the profile. The sharpest feature observed in the experiments had a FWHM of 190 nm. Given that the lateral resolution achievable with this instrument setup has been shown to be better than 20 nm (see Ref. **?**), the FWHM obtained was assumed to represent the resolution of the pattern under the limitations of the lensing effects already discussed in the main text.

Small spot low-energy electron diffraction (μ-LEED) measurements were collected from selected areas of 1.5 µm diameter using excitation energy 140 eV. Similar measurements were performed in several different regions across the diameter of each patterned feature, as well as in selected areas outside to verify that graphene had formed exclusively within the patterned regions. These measurements also confirmed the homogeneity of the newfound graphene layers.

Small-Area XPS and Work Function Measurements

Small area XPS and spatially resolved work function measurements were performed at the Bristol NanoESCA Facility, United Kingdom, to study the development of the patterned graphene on Ru with successive heat treatments and exposure to oxygen. XPS measurements of the C 1s, Ru 3d, Si 2p and O 1s core levels were performed using monochromatic Al Kα radiation ($h\nu = 1486.7$ eV) and an ARGUS CU analyzer, with an overall energy resolution of less than 300 meV. The development of the C 1s and Si 2p core levels at each relevant

stage of the experiment is showcased in Figures S3e and S3f, respectively.

The spatially resolved work functions (WFs) and associated SECs were recorded within the patterned graphene regions at 300 K using a NanoESCA II aberration corrected EF-PEEM equipped with a non-monochromatic Hg photoexcitation source ($h\nu \approx 5.2\,\mathrm{eV}$) and a 150 µm contrast aperture, using pass energy $E_\mathrm{P} = 50\,\mathrm{eV}$ and a 0.5 mm entrance slit. With the given settings, the instrument had a lateral resolution better than 150 nm and an overall energy resolution better than 150 meV. The NanoESCA allows accurate determination of the material work function ϕ_S using a fixed pass energy E_P, and tunable photoelectron retardation R and biasing of the sample under investigation. The energy of emitted electrons relative to the Fermi level $E - E_\mathrm{F}$ can be recorded according to the relation:

$$E - E_\mathrm{F} = h\nu - E_\mathrm{B} = R + E_\mathrm{P} + \phi_\mathrm{A} = E_\mathrm{K} + \phi_\mathrm{S}, \tag{1}$$

where $h\nu$ is the photoexcitation energy, E_B is the binding energy in the electron(s) photoexcited form the sample, E_K is their kinetic energy after being ejected to vacuum and ϕ_A is the analyzer work function. At zero E_K the electrons are unable to escape the sample, and ϕ_S is directly related to the measured energy position of the SEC relative to the Fermi level.[4]

Raman spectroscopy measurements

Following the iron-mediated graphitization of SiC at the UE-49-PGM-SMART endstation, the sample was removed from UHV and Raman mapping measurements were acquired using a HORIBA Jobin Yvon LabRAM HR Raman spectrometer at the Department of Physics, Aberystwyth University. A 10 mW 532 nm laser source was coupled to a x100 objective to form a 10 µm diameter circular spot on the G/Fe/SiC sample surface. A diffraction grating with 1800 lines/mm was selected, with confocal hole and slit widths of 1000 µm and 500 µm respectively.

To demonstrate the occurrence of graphene within the growth regions, Raman spectra

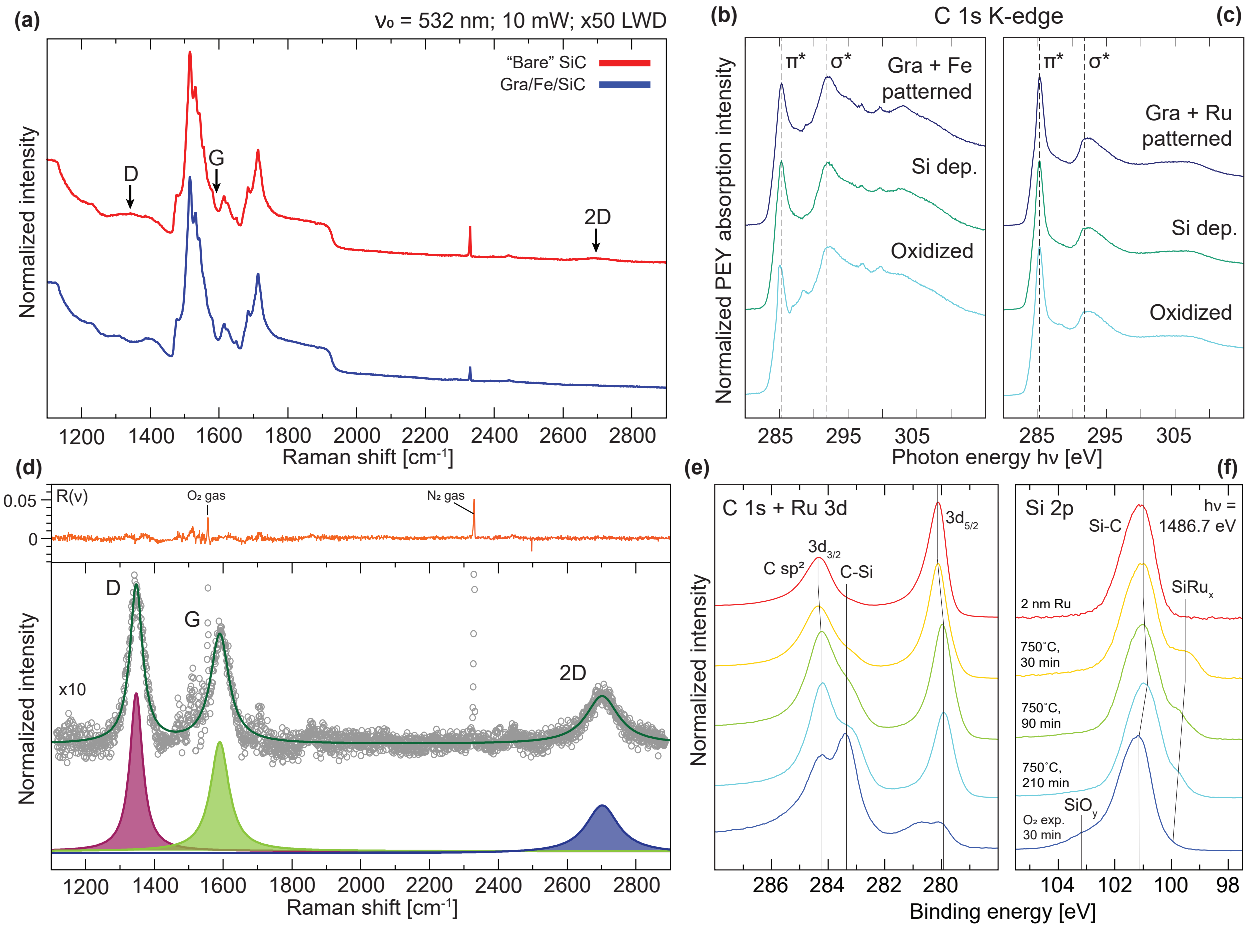

Figure S3: **Small-area Raman, NEXAFS and XPS measurements of patterned graphene. (a)** Raman spectroscopy measurements of SiC (red) and patterned graphene (500 µm circular regions) grown on a thin film of Fe on SiC (blue). Both excitation spectra were recorded using a 10 mW green (532 nm) laser with 10 µm spot size in separate regions of the sample; namely inside and outside of the graphene growth region. The approximate Raman shifts for the relevant vibrational modes excited, namely the D, G and 2D peaks, have been marked. **(b)** The near-edge X-ray absorption fine structure (NEXAFS) for graphene grown on patterned islands of Fe on SiC, before (dark blue) and after subsequent Si intercalation (green) and oxidation of the Si intercalants (light blue). **(c)** Similar NEXAFS measurements, but for graphene grown on patterned Ru on SiC. The measurements in both (b) and (c) were recorded using linearly polarized light at near grazing incidence ($\theta \approx 20°$) to the sample plane. **(d)** The difference spectrum of the two curves in (a), fitted using three Lorentzian peaks representing the three characteristic vibrational modes commonly seen in graphene Raman spectroscopy. The residual wave $R(v)$ bewtween the fit and the difference curve is also shown (top), together with annotations for additional excitations commonly observed in ambient systems. **(e,f)** X-ray photoelectron spectroscopy (XPS) measurements of the C 1s and Si 2p core levels, respectively, before (red) and after (yellow) deposition of 2 nm Ru, and with annealing to 750 °C for increasing duration (green) until substantial graphene formation is observed (light blue). The final two curves (dark blue) showcase the core levels after exposing the sample to oxygen at 200 mbar for 30 minutes.

with an energy window 1100-2900 cm^{-1} were recorded both inside the growth regions and outside, i.e. from the bare SiC substrate. Both spectra were normalized to the SiC feature at 1510 cm^{-1} (Figure S3a), and a difference spectrum was calculated between the two. The residual background was then fitted with a spline and that intensity was subtracted from each data set to remove any differences in the backgrounds from the SiC. The background corrected difference spectrum was finally fitted using Lorentzian peaks to obtain measures of the characteristic G, D and 2D peaks of the graphene as grown (Figure S3d).

To verify the spatial distribution of graphene exclusively inside the growth region, a Raman map with dimensions of 200 μm × 200 μm (10 μm step size, 400 spectra) was acquired, specifically centred on the edge of a graphene/Fe spot. The energy of the spectrometer was set around 2700 cm^{-1} (2500 to 2900 cm^{-1}) to record the characteristic 2D Raman mode indicative of ordered graphene/graphite at each point of the Raman map. Due to a lack of surface-enhancement effect for the Raman signal (i.e. usually observed for graphene on SiO_2/Si, see Refs. 5,6), extended acquisition times and multiple accumulations were required: a gate time of 120 seconds with 10 accumulations was used to improve the signal-to-noise ratio for peak fitting and remove noise from cosmic rays. Once recorded, similar background corrections and fittings as for the wide energy window were performed to obtain the overall intensity and FWHM of the graphene 2D peak as a function of its spatial location.

Synchrotron XPS and NEXAFS measurements

High-energy-resolution XPS and near edge X-ray absorption fine structure (NEXAFS) measurements were performed at the "Soft X-Ray" (SXR) endstation of the Australian Synchrotron (Melbourne, Victoria). The core levels C 1s, Si 2p, Fe 3p and Ru 3d were recorded at each relevant stage of the sample preparation, with excitation energies in the range 100-1200 eV using a Specs Phoibos 150 analyser with an overall energy resolution of 150-175 meV. The binding energy of each core level was calibrated to the metallic Fermi level position of

SiC with Fe or Ru added, over the corresponding energy range.

Upon graphene formation, NEXAFS measurements of the C 1s K-edge were performed at each successive stage of the experiment to assess the quality and stability of the graphene layers (see Figs. S3b and S3c). All measurements were performed in partial electron yield (PEY) mode and using linearly polarized light at grazing incidence angle ($\theta \approx 20°$) relative to the sample plane. During each measurement, a reference sample of HOPG was measured simultaenously with the sample for energy calibration of the spectra, whilst the photocurrent from a 50% transmissive gold mesh located just before the sample was used to measure the variation in photon flux during the scan. Additionally, the absolute photon intensities coming in to the analysis chamber were subsequently measured over the same energy range using a calibrated photodiode. The recorded NEXAFS spectra were normalized to the corresponding drain current on the Au mesh (for details, see Ref. 7), and the spectral response of the photodiode was used as a secondary normalization to correct for any carbon contamination on the Au mesh. Using the well known exciton resonance in HOPG occuring at 291.65 eV, the HOPG reference spectra measured in parallel with each scan were used to absolutely calibrate the energy scale of the data via a rigid shift. All energy and intensity flux corrections were performed using the Quick As NEXAFS Tool (QANT).[8]

Metal Diffusion into SiC

Diffusion of the metals Fe and Ru into the SiC substrate upon higher temperature annealing ($T > 700\,°C$) was ascertained by evaluating the relative intensity of Fe 3p and Ru 3d against the Si 2p signal of the substrate. By comparing the intensity of the metal to the underlying Si, any common attenuation of the two due to formation of carbon at the surface would, to a first approximation, be accounted for. As discussed in Ref. 3, consecutive heat treatments to 700 °C and then 800 °C of the gra/Fe/SiC and gra/Ru/SiC systems reduced the relative concentration of metal to SiC near the surface to $< 50\%$ of what was observed upon metallization.

An example of how the intensity of the Fe 3p core level of metallized SiC develops with temperature is shown in Figure S4. The Fe 3p signal at each stage has been scaled by the intensity of corresponding Si 2p signal, both recorded with $h\nu = 1254\,\text{eV}$ and with any differences in photoelectron inelastic mean-free paths (IMFPs) and photo-ionization cross-sections corrected for.

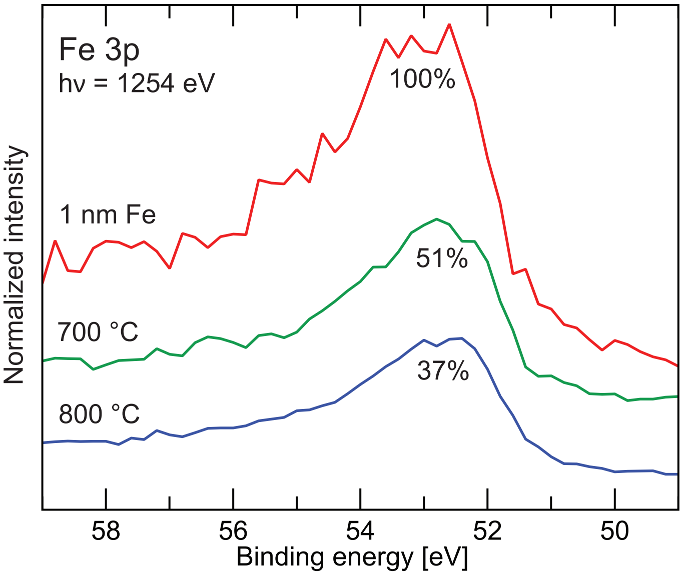

Figure S4: Development of the Fe 3p core level for metallized 6H-SiC with increasing temperature. The intensity of each Fe 3p peak has been normalized to the Si 2p intensity at the same experimental stage, corrected for differences in IMFPs and photo-ionization cross-sections. Annealing above 700 °C reduces the concentration of Fe near the surface by $> 60\%$.

References

(1) Cooil, S.; Song, F.; Williams, G.; Roberts, O.; Langstaff, D.; Jørgensen, B.; Høydalsvik, K.; Breiby, D.; Wahlström, E.; Evans, D.; Wells, J. Iron-Mediated Growth of Epitaxial Graphene on SiC and Diamond. *Carbon* **2012**, *50*, 5099–5105.

(2) Cooil, S.; Wells, J.; Hu, D.; Niu, Y.; Zakharov, A.; Bianchi, M.; Evans, D. Controlling the Growth of Epitaxial Graphene on Metalized Diamond (111) Surface. *Appl. Phys. Lett.* **2015**, *107*, 181603.

(3) Røst, H. I.; Chellappan, R. K.; Strand, F. S.; Grubišić-Čabo, A.; Reed, B. P.; Prieto, M. J.; Tănase, L. C.; Caldas, L. d. S.; Wongpinij, T.; Euaruksakul, C.; Schmidt, T.; Tadich, A.; Cowie, B. C. C.; Li, Z.; Cooil, S. P.; Wells, J. W. Low-Temperature Growth of Graphene on a Semiconductor. *J. Phys. Chem. C* **2021**, *125*, 4243–4252.

(4) Escher, M.; Weber, N.; Merkel, M.; Krömker, B.; Funnemann, D.; Schmidt, S.; Reinert, F.; Forster, F.; Hüfner, S.; Bernhard, P.; Ziethen, C.; Elmers, H.; Schönhense, G. NanoESCA: Imaging UPS and XPS with High Energy Resolution. *J. Electron Spectros. Relat. Phenomena* **2005**, *144-147*, 1179–1182, Proceeding of the Fourteenth International Conference on Vacuum Ultraviolet Radiation Physics.

(5) Wang, Y.; Ni, Z.; Shen, Z.; Wang, H.; Wu, Y. Interference Enhancement of Raman Signal of Graphene. *Applied Physics Letters* **2008**, *92*, 043121.

(6) Yoon, D.; Moon, H.; Son, Y.-W.; Choi, J. S.; Park, B. H.; Cha, Y. H.; Kim, Y. D.; Cheong, H. Interference Effect on Raman Spectrum of Graphene on SiO_2/Si. *Phys. Rev. B* **2009**, *80*, 125422.

(7) Watts, B.; Thomsen, L.; Dastoor, P. Methods in Carbon K-Edge NEXAFS: Experiment and Analysis. *Journal of Electron Spectroscopy and Related Phenomena* **2006**, *151*, 105–120.

(8) Gann, E.; McNeill, C. R.; Tadich, A.; Cowie, B. C. C.; Thomsen, L. *Quick AS NEXAFS Tool* (*QANT*): A Program for NEXAFS Loading and Analysis Developed at the Australian Synchrotron. *J. Synchrotron Rad.* **2016**, *23*, 374–380.
